# Supplementary material for: Similarities and differences in Alzheimer’s dementia comorbidities in racialized populations identified from electronic medical records
Source: Commun Med (Lond). 2023 Apr 8;3:50. doi: 10.1038/s43856-023-00280-2 (PMC10082816; doi:10.1038/s43856-023-00280-2)
Supplement: Supplementary file 1 — Supplementary Material [file 43856_2023_280_MOESM1_ESM.pdf]

Similarities and Differences in Alzheimer's Dementia Comorbidities in Racialized Populations Identified from Electronic Medical Records

Supplementary Material

Sarah Woldemariam[1], Alice Tang[1,2], Tomiko Oskotsky[1,3], Kristine Yaffe[4], Marina Sirota[1,3*]

[1]Bakar Computational Health Sciences Institute, University of California San Francisco, San Francisco, California, USA; [2]School of Medicine, University of California San Francisco, San Francisco, California, USA; [3]Department of Pediatrics, University of California San Francisco, San Francisco, California, USA; [4]Department of Psychiatry and Behavioral Sciences, University of California San Francisco, San Francisco, California, USA

*Corresponding author; Marina.Sirota@ucsf.edu

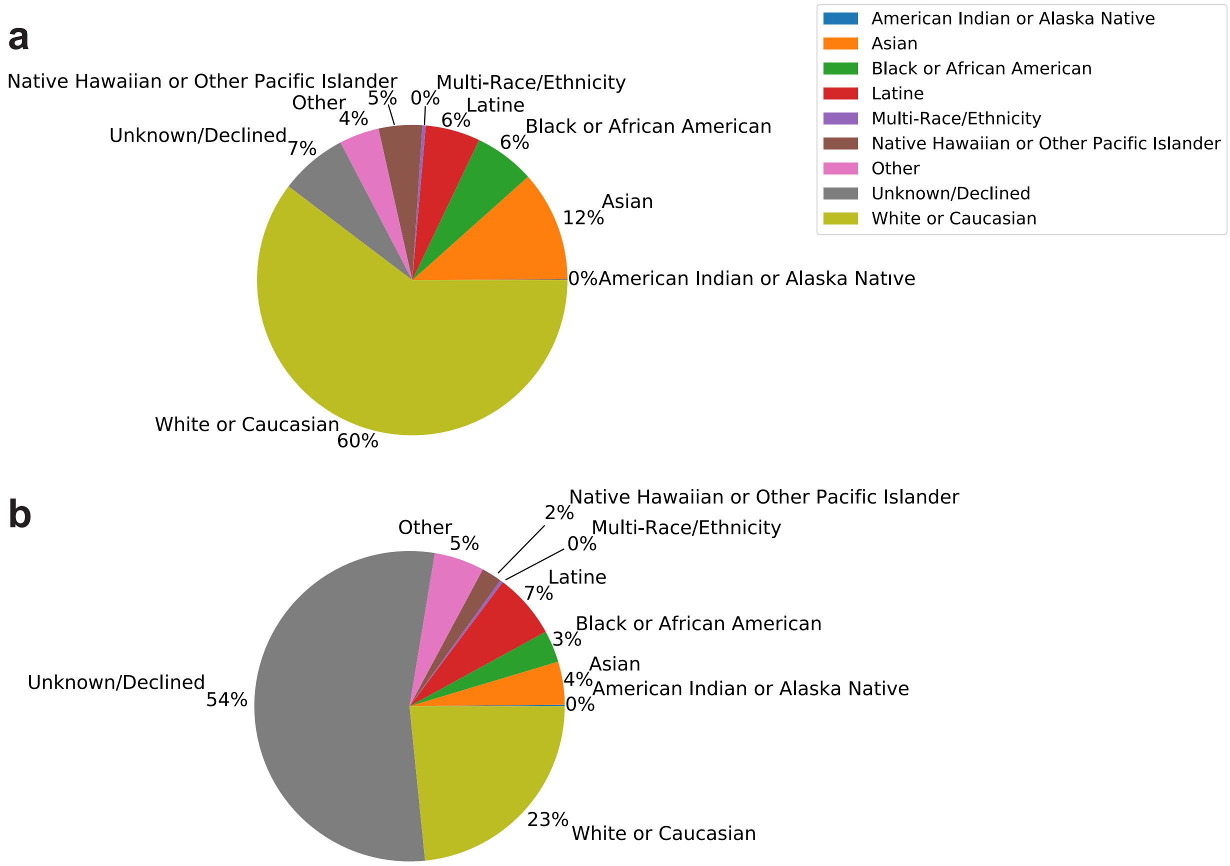

Supplementary Figure 1: Race and ethnicity categories of patients at UCSF.
a Race and ethnicity categories of patients with AD at UCSF.
b Race and ethnicity categories of all patients at UCSF.

blue = American Indian or Alaska Native; orange = Asian; dark green = Black or African American; purple = Multi-Race/Ethnicity; brown = Native Hawaiian or Other Pacific Islander; pink = Other; gray = Unknown/Declined; light green = White or Caucasian. AD = Alzheimer’s dementia; UCSF = University of California San Francisco.

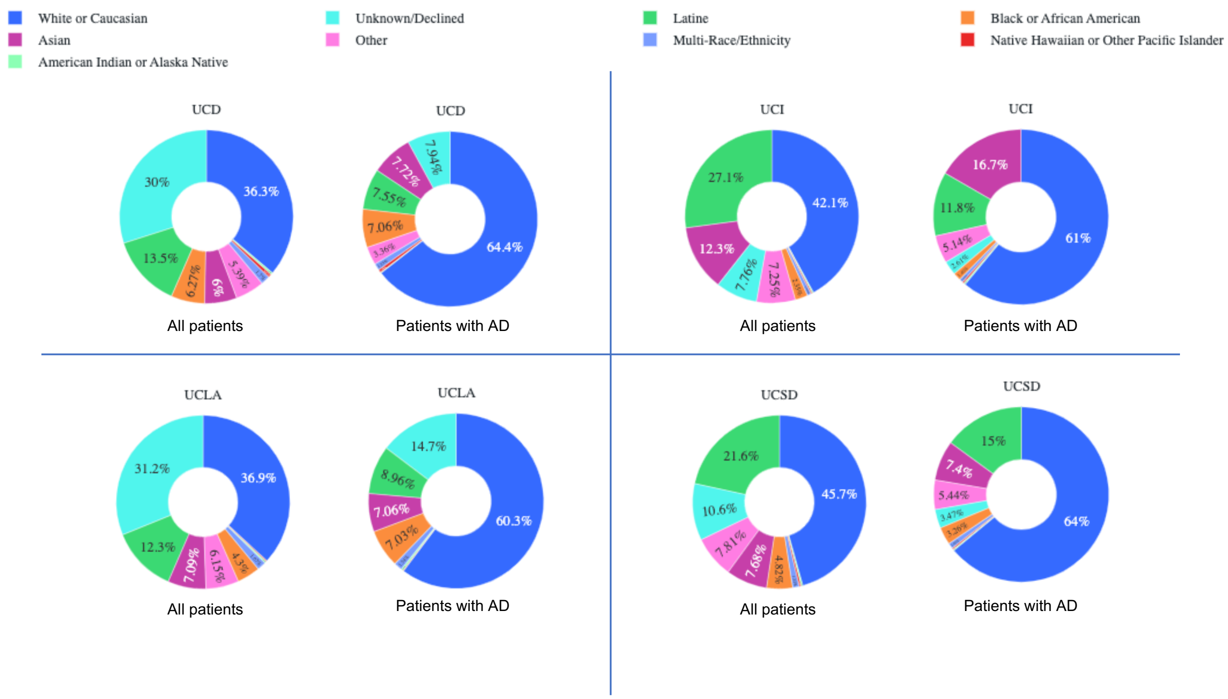

Supplementary Figure 2: Demographics of all patients, as well as patients with AD, at each UC health center in the UC-wide validation cohort.

All patients, as well as all patients diagnosed with non early-stage AD, at each University of California (UC) health center in the UC-wide validation cohort.

UCD = UC Davis; UCI = UC Irvine; UCLA = UC Los Angeles; UCSD = UC San Diego; AD = Alzheimer’s dementia.

dark purple = White or Caucasian; cyan = Unknown/Declined; dark green = Latine; orange = Black or African American; magenta = Asian; pink = Other; light purple = Multi-Race/Ethnicity; red = Native Hawaiian or Pacific Islander; light green = American Indian or Alaska Native.

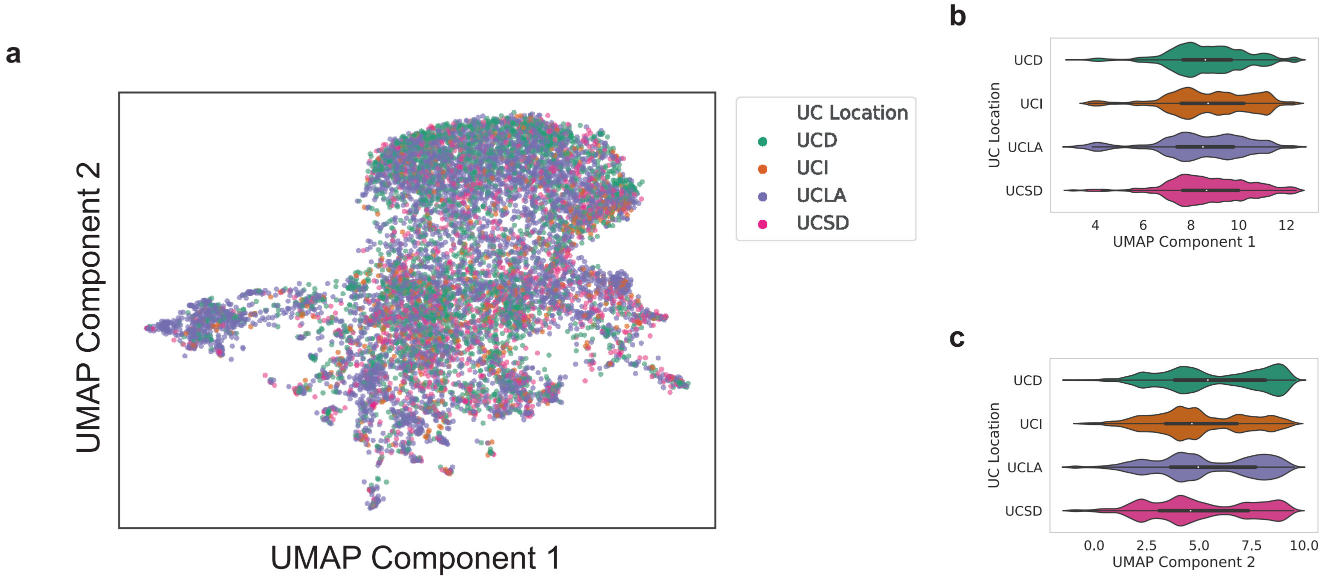

Supplementary Figure 3: UMAP of patients' phenotypic profiles based on UC location.

a UMAP of patients' phenotypic profiles UC-wide, based on UC location.
b Distribution of patients along the first UMAP component, based on UC location.
c Distribution of patients along the second UMAP component, based on UC location.

Data were derived from n = 10,971 patients in the UC-wide validation cohort.

UMAP = Uniform Manifold Approximation and Projection.

green = University of California Davis (UCD); orange = University of California Irvine (UCI); purple = University of California Los Angeles (UCLA); pink = University of California San Diego (UCSD).

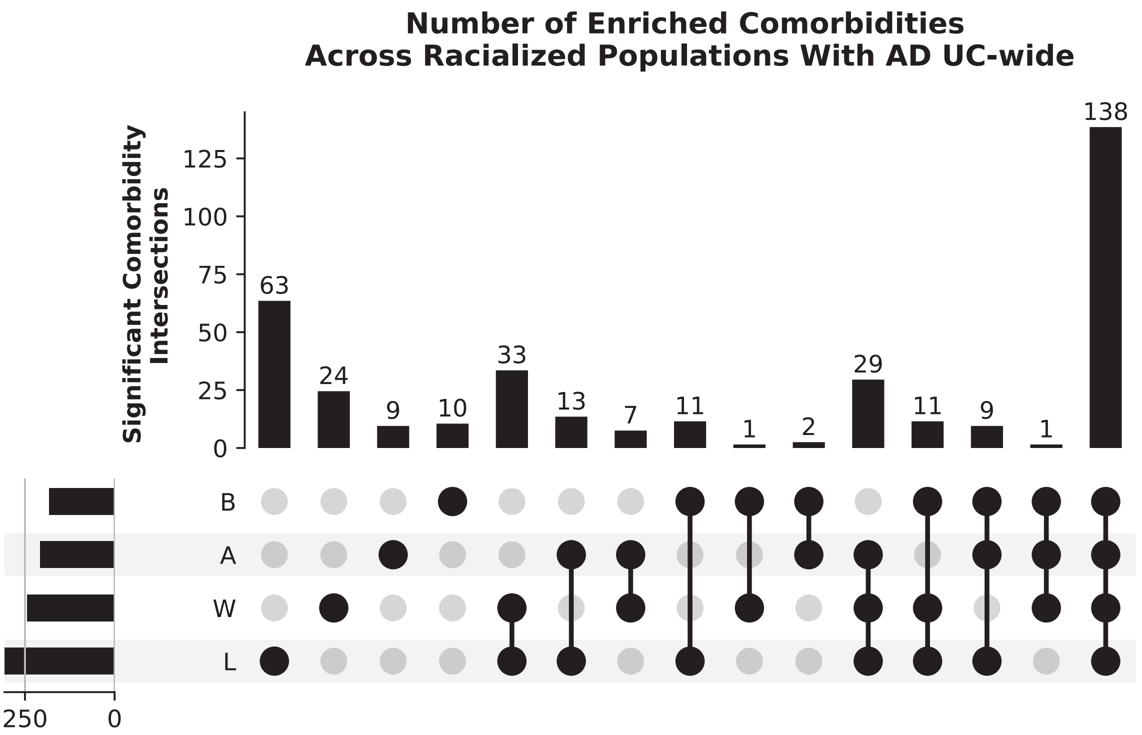

Supplementary Figure 4: Most comorbidities significant for all racialized populations at UCSF are validated in the UC-wide validation cohort.

Upset plot of significant comorbidity intersections across racialized populations. Rows indicate each racialized population analyzed. Bar chart shows single and overlapping significant phenotypes across racialized populations. Statistical significance was determined using Fisher's exact or chi-squared tests, comparing 896 phenotypes between patients with AD (n = 994) and control patients (n = 1,988) within each racialized population. Significance corresponded to a Bonferroni-corrected p-value < 0.05. A = Asian-identified patients; B = Black-identified patients; L = Latine-identified patients; W = White-identified patients; AD = Alzheimer's dementia; UCSF = University of California San Francisco.

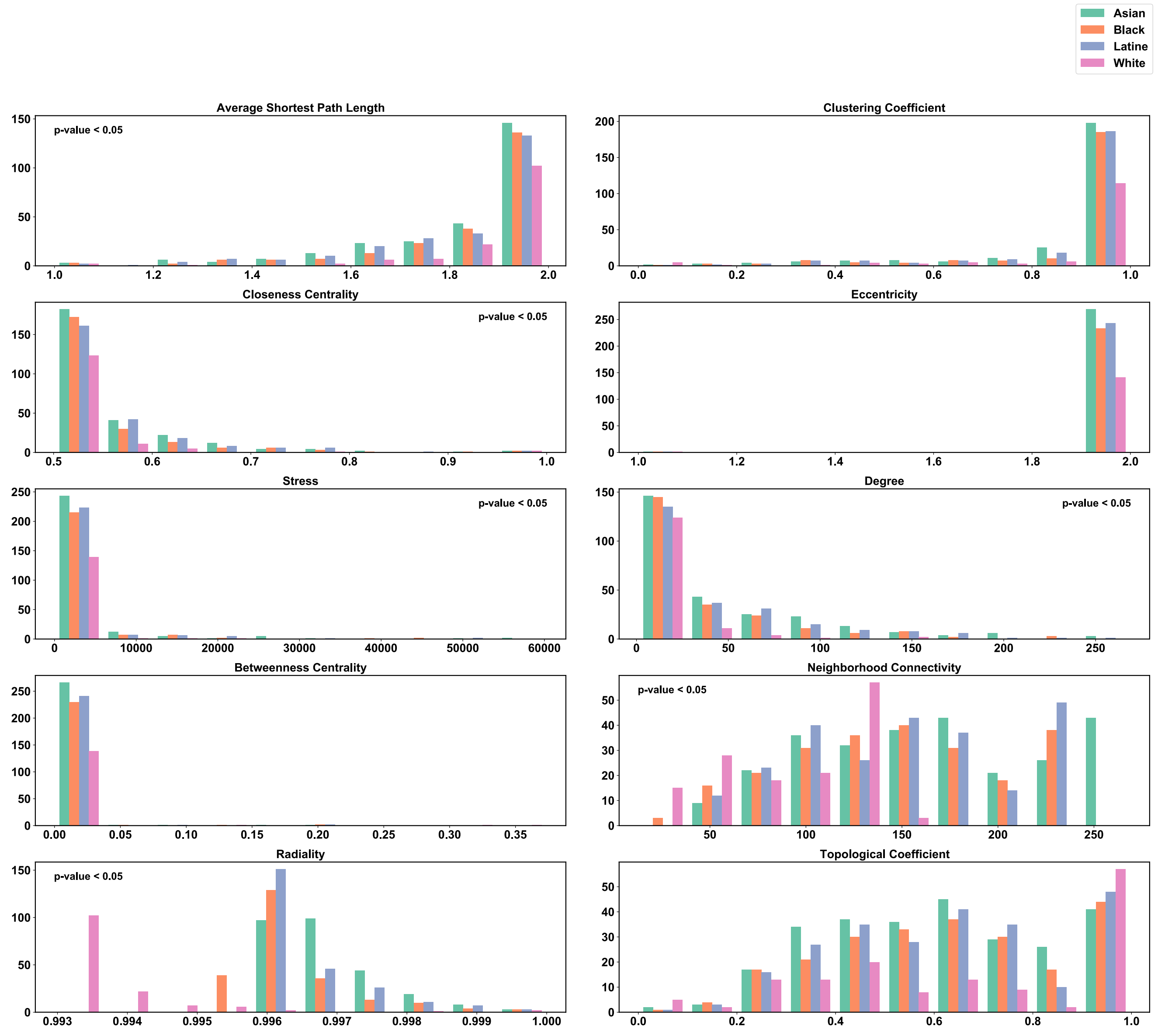

Supplementary Figure 5: Network metric distributions for UCSF-based AD phenotype networks are stratified by identified R&E.

At least 5% of Asian-identified patients with AD shared 270 nodes; at least 5% of Black-identified patients with AD shared 234 nodes; at least 5% of Latine-identified patients with AD shared 244 nodes; and at least 5% of White-identified patients with AD shared 142 nodes. n = 422 patients with AD per identified R&E category.

green = Asian-identified; orange = Black-identified; blue = Latine-identified; pink = White-identified; AD = Alzheimer’s dementia; R&E = race and ethnicity; UCSF = University of California San Francisco.

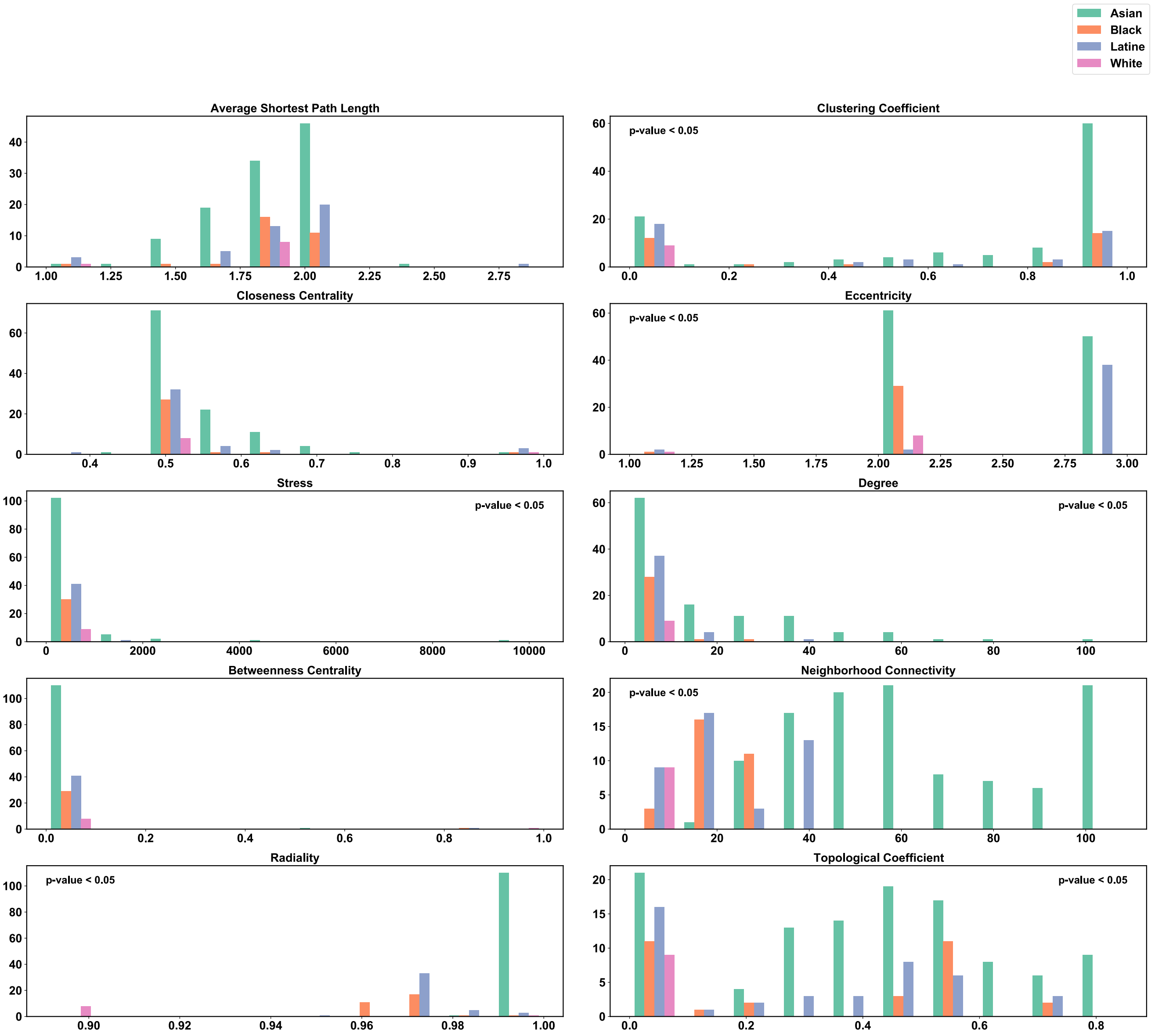

Supplementary Figure 6: Network metric distributions for UCSF-based control phenotype networks are stratified by identified R&E.

At least 5% of Asian-identified control patients shared 111 nodes; at least 5% of Black-identified control patients shared 30 nodes; at least 5% of Latine-identified control patients shared 42 nodes; and at least 5% of White-identified control patients shared 9 nodes. n = 844 control patients per identified R&E category.

green = Asian-identified; orange = Black-identified; blue = Latine-identified; pink = White-identified; AD = Alzheimer's dementia; R&E = race and ethnicity; UCSF = University of California San Francisco.
