# Supplementary material for: Similarities and differences in Alzheimer’s dementia comorbidities in racialized populations identified from electronic medical records
Source: Commun Med (Lond). 2023 Apr 8;3:50. doi: 10.1038/s43856-023-00280-2 (PMC10082816; doi:10.1038/s43856-023-00280-2)
Supplement: Supplementary file 14 — Reporting Summary [file 43856_2023_280_MOESM14_ESM.pdf]

nature research

Corresponding author(s): Dr. Marina Sirota

Last updated by author(s): Mar 13, 2023

Reporting Summary

Nature Research wishes to improve the reproducibility of the work that we publish. This form provides structure for consistency and transparency in reporting. For further information on Nature Research policies, see our Editorial Policies and the Editorial Policy Checklist.

Statistics

For all statistical analyses, confirm that the following items are present in the figure legend, table legend, main text, or Methods section.

n/a	Confirmed	
☐	☒	The exact sample size (n) for each experimental group/condition, given as a discrete number and unit of measurement
☒	☐	A statement on whether measurements were taken from distinct samples or whether the same sample was measured repeatedly
☐	☒	The statistical test(s) used AND whether they are one- or two-sided<br>*Only common tests should be described solely by name; describe more complex techniques in the Methods section.*
☒	☐	A description of all covariates tested
☐	☒	A description of any assumptions or corrections, such as tests of normality and adjustment for multiple comparisons
☐	☒	A full description of the statistical parameters including central tendency (e.g. means) or other basic estimates (e.g. regression coefficient) AND variation (e.g. standard deviation) or associated estimates of uncertainty (e.g. confidence intervals)
☐	☒	For null hypothesis testing, the test statistic (e.g. F, t, r) with confidence intervals, effect sizes, degrees of freedom and P value noted<br>*Give P values as exact values whenever suitable.*
☒	☐	For Bayesian analysis, information on the choice of priors and Markov chain Monte Carlo settings
☐	☒	For hierarchical and complex designs, identification of the appropriate level for tests and full reporting of outcomes
☐	☒	Estimates of effect sizes (e.g. Cohen's d, Pearson's r), indicating how they were calculated

Our web collection on statistics for biologists contains articles on many of the points above.

Software and code

Policy information about availability of computer code

Data collection	No new data was collected for this study. No software was used for data collection.
Data analysis	See Methods

For manuscripts utilizing custom algorithms or software that are central to the research but not yet described in published literature, software must be made available to editors and reviewers. We strongly encourage code deposition in a community repository (e.g. GitHub). See the Nature Research guidelines for submitting code & software for further information.

Data

Policy information about availability of data

All manuscripts must include a data availability statement. This statement should provide the following information, where applicable:

- Accession codes, unique identifiers, or web links for publicly available datasets
- A list of figures that have associated raw data
- A description of any restrictions on data availability

The de-identified UCSF EHR database is available to UCSF-affiliated individuals who can contact UCSF's Clinical and Translational Science Institute (CTSI) (ctsi@ucsf.edu) or the UCSF's Information Commons team for more information (Info.Commons@ucsf.edu). If the reader is unaffiliated with UCSF, they can set up an official collaboration with a UCSF-affiliated investigator by contacting the PI, Marina Sirota (marina.sirota@ucsf.edu). Requests should be processed within a couple of weeks. UCDDP is only available to UC researchers who have completed analyses in their respective UC first and have provided justification for scaling their analyses across UC health centers.

Field-specific reporting

Please select the one below that is the best fit for your research. If you are not sure, read the appropriate sections before making your selection.

☒ Life sciences ☐ Behavioural & social sciences ☐ Ecological, evolutionary & environmental sciences

For a reference copy of the document with all sections, see nature.com/documents/nr-reporting-summary-flat.pdf

Life sciences study design

All studies must disclose on these points even when the disclosure is negative.

Sample size	No sample size calculation was performed prior to the study. Sample size was determined by the number of patients with Alzheimer's disease in the UCSF (7,409; after two rounds of propensity score matching, 1,688) and UCDDP (19,686; after two rounds of propensity score matching, 3,976) EMR databases (for UCDDP specifically, the number of patients with Alzheimer's disease who received care at either UCD, UCI, UCLA, and UCSD. A 1 to 2 matching ratio was utilized to identify controls in order to ensure sufficient power to capture common comorbidities in the population.
Data exclusions	No data excluded from analysis, but cohort comprised patients over 64 years old in order to capture late-onset Alzheimer's disease.
Replication	Not applicable, this study did not acquire new data. Replication of analyses were performed in the UCDDP.
Randomization	Not applicable, this study did not acquire new data.
Blinding	Not applicable, this study did not acquire new data.

Reporting for specific materials, systems and methods

We require information from authors about some types of materials, experimental systems and methods used in many studies. Here, indicate whether each material, system or method listed is relevant to your study. If you are not sure if a list item applies to your research, read the appropriate section before selecting a response.

Materials & experimental systems

n/a	Involved in the study	
☒	☐	Antibodies
☒	☐	Eukaryotic cell lines
☒	☐	Palaeontology and archaeology
☒	☐	Animals and other organisms
☐	☒	Human research participants
☒	☐	Clinical data
☒	☐	Dual use research of concern

Methods

n/a	Involved in the study	
☒	☐	ChIP-seq
☒	☐	Flow cytometry
☒	☐	MRI-based neuroimaging

Human research participants

Policy information about studies involving human research participants

Population characteristics	Study participants were identified from de-identified UCSF and UCDDP EMR databases, which includes patients who received care at UCSF or, in the case of UCDDP, either UCD, UCI, UCLA, or UCSD. The cohorts include patients over 64 years of age. Propensity score matched control participants were also identified from the UCSF and UCDDP EMR databases. Demographic characteristics are shown in Table 1.
Recruitment	No recruitment was performed for this study.
Ethics oversight	Analysis of UCSF de-identified EMR data was performed by UCSF employees under Institutional Review Board approval (IRB Study Number 20-32422). Analysis of UC-wide de-identified EMR data via the UC Data Discovery Portal was performed by UCSF employees under approval by the UC Health IRBs for research use. Since only de-identified data was analyzed, written informed consent was waived by all UC institutions.

Note that full information on the approval of the study protocol must also be provided in the manuscript.
