# Supplementary material for: Similarities and differences in Alzheimer’s dementia comorbidities in racialized populations identified from electronic medical records
Source: Commun Med (Lond). 2023 Apr 8;3:50. doi: 10.1038/s43856-023-00280-2 (PMC10082816; doi:10.1038/s43856-023-00280-2)
Supplement: Supplementary file 15 — Description of Additional Supplementary Files [file 43856_2023_280_MOESM15_ESM.pdf]

Description of Additional Supplementary Files

File Name: Supplementary Data 1

Description: UMAP components' means and standard deviations. UCSF_AD sheet contains UCSF UMAP components' means and standard deviations based on Alzheimer's dementia status; UCSF_RE sheet contains UCSF UMAP components' means and standard deviations based on identified race and ethnicity; UC-wide_AD sheet contains UC-wide UMAP components' means and standard deviations based on Alzheimer's dementia status; UC-wide_RE sheet contains UC-wide UMAP components' means and standard deviations based on identified race and ethnicity; UC-wide_UCLocation sheet contains UC-wide UMAP components' means and standard deviations based on UC Location.

File Name: Supplementary Data 2

Description: Phenotype Differential Analysis, UCSF. Results from Phenotype Differential Analysis, including p-value of statistical test and odds ratio. phenotype = Phecode-corresponding phenotype, mapped from ICD diagnosis; pvalue_RE* = Fisher's exact or chi-squared test p-value; if below Bonferroni-corrected p-value < 0.05, phenotype is significantly associated with either (1) patients with AD relative to controls or (2) control patients relative to patients with AD; -log_pvalue_RE* = - log10 of the p-value; Count_AD_RE* = Number of patients with AD with phenotype; Count_con_RE* = Number of control patients with phenotype; OddsRatio_RE* = Odds ratio. If p-value is significant and odds ratio is greater than 1, phenotype associated with patients with AD; If p-value is significant and odds ratio is less than 1, phenotype is associated with control patients; OddsRatio_CI_lb_RE* = Lower bound of the 95% confidence interval for the odds ratio; OddsRatio_CI_ub_RE* = Upper bound of the 95% confidence interval for the odds ratio; log2_oddsratio_RE* = log 2 of the odds ratio; icd10_chapter = Corresponds to phecode category of phenotype, which is based on ICD-10-CM chapters; Sig_RE* = Whether phenotype reached significance; OddsRatio_RE_AD* = Whether phenotype is associated with patients with AD, assessed by whether OddsRatio_RE > 1; Sig_RE_AD* = Whether phenotype is significantly associated with patients with AD; Significance (Supplementary Data File 2 only) = Racialized populations for which the phenotype is significantly associated for patients with AD. *RE can correspond to (1) A (Asian-identified patients); (2) B (Black-identified patients); (3) L (Latine-identified patients); (4) W (White-identified patients).

File Name: Supplementary Data 3

Description: Phenotype Differential Analysis, UC-wide validation cohort. Results from Phenotype Differential Analysis, including p-value of statistical test and odds ratio. phenotype = Phecode-corresponding phenotype, mapped from ICD diagnosis; pvalue_RE* = Fisher's exact or chi-squared test p-value; if below Bonferroni-corrected p-value < 0.05, phenotype is significantly associated with either (1) patients with AD relative to controls or (2) control patients relative to patients with AD; -log_pvalue_RE* = - log10 of the p-value; Count_AD_RE* = Number of patients with AD with phenotype; Count_con_RE* = Number of control patients with phenotype; OddsRatio_RE* = Odds ratio. If p-value is significant and odds ratio is greater

than 1, phenotype associated with patients with AD; If p-value is significant and odds ratio is less than 1, phenotype is associated with control patients; OddsRatio_CI_lb_RE* = Lower bound of the 95% confidence interval for the odds ratio; OddsRatio_CI_ub_RE* = Upper bound of the 95% confidence interval for the odds ratio; log2_oddsratio_RE* = log 2 of the odds ratio; icd10_chapter = Corresponds to phecode category of phenotype, which is based on ICD-10-CM chapters; Sig_RE* = Whether phenotype reached significance; OddsRatio_RE_AD* = Whether phenotype is associated with patients with AD, assessed by whether OddsRatio_RE > 1; Sig_RE_AD* = Whether phenotype is significantly associated with patients with AD; Significance (Supplementary Data File 2 only) = Racialized populations for which the phenotype is significantly associated for patients with AD. *RE can correspond to (1) A (Asian-identified patients); (2) B (Black-identified patients); (3) L (Latine-identified patients); (4) W (White-identified patients).

File Name: Supplementary Data 4

Description: Network Analysis, Node Tables, UCSF. phenotype = Phecode-corresponding phenotype, mapped from ICD diagnosis; icd10_chapter = Corresponds to phecode category of phenotype, which is based on ICD-10-CM chapters; RE = Number of patients with the phenotype. RE could correspond to: (1) Asian; (2) Black; (3) Latine; (4) White; pRE = Percentage of patients with the phenotype. pRE could correspond to: (1) pA - percentage of Asian-identified patients; (2) pB - percentage of Black-identified patients; (3) pL - percentage of Latine-identified patients; (4) pW - percentage of White-identified patients; AverageShortestPathLength = Measure of phenotype's (i.e. node's) average shortest path length; ClusteringCoefficient = Measure of phenotype's (i.e. node's) clustering coefficient; ClosenessCentrality = Measure of phenotype's (i.e. node's) closeness centrality; Eccentricity = Measure of phenotype's (i.e. node's) eccentricity; Stress = Measure of phenotype's (i.e. node's) stress; Degree = Measure of phenotype's (i.e. node's) degree; BetweennessCentrality = Measure of phenotype's (i.e. node's) betweenness centrality; NeighborhoodConnectivity = Measure of phenotype's (i.e. node's) neighborhood connectivity; Radiality = Measure of phenotype's (i.e. node's) radiality; TopologicalCoefficient = Measure of phenotype's (i.e. node's) topological coefficient.

File Name: Supplementary Data 5

Description: Network Analysis, Edge Tables, UCSF. phenotype_1 = 1 of 2 phecode-corresponding phenotypes in the pair, mapped from ICD diagnosis. Phenotype pair corresponds to an undirected edge; phenotype_2 = 2 of 2 phecode-corresponding phenotypes in the pair, mapped from ICD diagnosis. Phenotype pair corresponds to an undirected edge; RE = Number of patients with the phenotype pair. RE could correspond to: (1) Asian; (2) Black; (3) Latine; (4) White; pRE = Percentages of patients with the phenotype pair. pRE could correspond to: (1) pA - percentage of Asian-identified patients; (2) pB - percentage of Black-identified patients; (3) pL - percentage of Latine-identified patients; (4) pW - percentage of White-identified patients.

File Name: Supplementary Data 6

Description: Network metric means and standard deviations. UCSF_AD_Networks sheet contains network metrics' means and standard deviations for UCSF AD networks, stratified by identified race and ethnicity; UCSF_Control_Networks sheet contains network metrics' means and standard deviations for UCSF control networks, stratified by identified race and ethnicity; UC-wide_AD_Networks sheet contains network metrics' means and standard deviations for UC-wide AD networks, stratified by identified race and ethnicity; UC-wide_Control_Networks sheet contains network metrics' means and standard deviations for UC-wide control networks, stratified by identified race and ethnicity.

File Name: Supplementary Data 7

Description: Mann-Whitney *U* test statistics and p-values comparing AD networks and matched control networks at UCSF and UC-wide. Identified Race and Ethnicity - RE - statistic = Mann-Whitney U test statistic for given network metric comparing RE-identified AD network. RE may correspond to Asian, Black, Latine, or White; Identified Race and Ethnicity - RE - p-value = Mann-Whitney U p-value for given network metric comparing RE-identified AD network and RE-identified control network. RE may correspond to Asian, Black, Latine, or White; 'network metric' = node-level network 'network metric' compared between AD networks and control networks. 'network metric' may correspond to average shortest path length, closeness centrality, clustering coefficient, degree, eccentricity, neighborhood connectivity, radiality, stress, or topological coefficient; * = network metrics significantly different between AD and control networks for all racialized populations at UCSF and UC-wide.

File Name: Supplementary Data 8

Description: Top phenotype pairs shared between UCSF and the UC-wide validation cohort for patients with AD, stratified by identified R&E. UCSF (%) = Percentage of AD patients with phenotype pair at UCSF; UC-wide (%) = Percentage of AD patients with phenotype pair UC-wide; * = phenotype pairs shared for all identified R&E; ** = phenotype pairs specific to identified R&E; AD = Alzheimer's dementia; R&E = identified race and ethnicity.

File Name: Supplementary Data 9

Description: Top phenotype pairs shared between UCSF and the UC-wide validation cohort for control patients, stratified by identified R&E. UCSF (%) = Percentage of control patients with phenotype pair at UCSF; UC-wide (%) = Percentage of control patients with phenotype pair UC-wide; * = phenotype pairs shared for all identified R&E; ** = phenotype pairs specific to identified R&E; AD = Alzheimer's dementia; R&E = identified race and ethnicity.

File Name: Supplementary Data 10

Description: Network Analysis, Node Tables, UC-wide validation cohort. phenotype = Phecode-corresponding phenotype, mapped from ICD diagnosis; icd10_chapter = Corresponds to phecode category of phenotype, which is based on ICD-10-CM chapters; RE = Number of patients with the phenotype. RE could correspond to: (1) Asian; (2) Black; (3) Latine; (4) White; pRE = Percentage of patients with the phenotype. pRE could correspond to:

(1) pA - percentage of Asian-identified patients; (2) pB - percentage of Black-identified patients; (3) pL - percentage of Latine-identified patients; (4) pW - percentage of White-identified patients; AverageShortestPathLength = Measure of phenotype's (i.e. node's) average shortest path length; ClusteringCoefficient = Measure of phenotype's (i.e. node's) clustering coefficient;
ClosenessCentrality = Measure of phenotype's (i.e. node's) closeness centrality; Eccentricity = Measure of phenotype's (i.e. node's) eccentricity; Stress = Measure of phenotype's (i.e. node's) stress; Degree = Measure of phenotype's (i.e. node's) degree; BetweennessCentrality = Measure of phenotype's (i.e. node's) betweenness centrality; NeighborhoodConnectivity = Measure of phenotype's (i.e. node's) neighborhood connectivity; Radiality = Measure of phenotype's (i.e. node's) radiality; TopologicalCoefficient = Measure of phenotype's (i.e. node's) topological coefficient.

File Name: Supplementary Data 11

Description: Network Analysis, Edge Tables, UC-wide validation cohort. phenotype_1 = 1 of 2 phecode-corresponding phenotypes in the pair, mapped from ICD diagnosis. Phenotype pair corresponds to an undirected edge; phenotype_2 = 2 of 2 phecode-corresponding phenotypes in the pair, mapped from ICD diagnosis. Phenotype pair corresponds to an undirected edge; RE = Number of patients with the phenotype pair. RE could correspond to: (1) Asian; (2) Black; (3) Latine; (4) White; pRE = Percentages of patients with the phenotype pair. pRE could correspond to: (1) pA - percentage of Asian-identified patients; (2) pB - percentage of Black-identified patients; (3) pL - percentage of Latine-identified patients; (4) pW - percentage of White-identified patients.
